# Palliative care needs of people and/or their families with serious and/or chronic health conditions in low- or middle-income country (LMIC) humanitarian settings—a systematic scoping review protocol

**DOI:** 10.1186/s13643-024-02521-4

**Published:** 2024-04-11

**Authors:** Michelle McGannan, Liz Grant, David Fearon, Marshall Dozier, Victoria Barber-Fleming

**Affiliations:** 1 Deanery of Molecular, Genetic and Population Health Sciences, Usher Institute, College of Medicine and Veterinary Medicine, University of Edinburgh, Edinburgh, UK; 2 Global Health and Development—Centre for Global Health, Deanery of Molecular, Genetic and Population Health Sciences, Usher Institute, College of Medicine and Veterinary Medicine, University of Edinburgh, Edinburgh, UK; 3 Library Academic Support, University of Edinburgh, Edinburgh, UK; 4 Advanced Care Research Centre (ACRC), College of Medicine and Veterinary Medicine and School of Engineering, University of Edinburgh, Edinburgh, UK

**Keywords:** Palliative care, Palliative care needs, Humanitarian, Low-middle-income countries (LMIC), Chronic health conditions

## Abstract

**Background:**

Palliative care in low- or middle-income country (LMIC) humanitarian settings is a new area, experiencing a degree of increased momentum over recent years. The review contributes to this growing body of knowledge, in addition to identifying gaps for future research. The overall aim is to systematically explore the evidence on palliative care needs of patients and/or their families in LMIC humanitarian settings.

**Methods:**

Arksey and O’Malley’s (Int J Soc Res Methodol. 8:19-32, 2005) scoping review framework forms the basis of the study design, following further guidance from Levac et al. (Implement Sci 5:1-9, 2010), the Joanna Briggs Institute (JBI) Peters et al. (JBI Reviewer’s Manual JBI: 406-452, 2020), and the Preferred Reporting Items for Systematic reviews and Meta-Analyses extension for Scoping Reviews (PRISMA-ScR) from Tricco et al. (Ann Intern Med 169:467-73, 2018). This incorporates a five-step approach and the population, concept, and context (PCC) framework. Using already identified key words/terms, searches for both published research and gray literature from January 2012 to October 2022 will be undertaken using databases (likely to include Cumulative Index of Nursing and Allied Health (CINAHL), MEDLINE, Embase, Global Health, Scopus, Applied Social Science Index and Abstracts (ASSIA), Web of Science, Policy Commons, JSTOR, Library Network International Monetary Fund and World Bank, Google Advanced Search, and Google Scholar) in addition to selected pre-print sites and websites. Data selection will be undertaken based on the inclusion and exclusion criteria and will be reviewed at each stage by two reviewers, with a third to resolve any differences. Extracted data will be charted in a table. Ethical approval is not required for this review.

**Discussion:**

Findings will be presented in tables and diagrams/charts, followed by a narrative description. The review will run from late October 2022 to early 2023. This is the first systematic scoping review specifically exploring the palliative care needs of patients and/or their family, in LMIC humanitarian settings. The paper from the review findings will be submitted for publication in 2023.

**Supplementary Information:**

The online version contains supplementary material available at 10.1186/s13643-024-02521-4.

## Background

An estimated 274 million people are in need of humanitarian protection and assistance in 2022 [1], and while palliative care has rarely been undertaken in these situations [2], since 2016 a recognition of the need for palliative care integration into humanitarian settings has been growing [3] with the importance of this becoming increasingly recognized [4]. Palliative care has been incorporated into the Humanitarian Sphere Handbook [5], in addition to the WHO publication: “Integrating palliative care and symptom relief into the response to humanitarian crises and emergencies” in 2018 [2]. The first *Field Manual for Palliative Care in Humanitarian Crises* was published in 2020 [6].

The growing momentum in this area includes an ever-expanding body of literature, ranging across the spectrum from primary research studies to reports and anecdotal reflections. Since 2017, a small number of systematic or scoping reviews have been undertaken — these have included studies focusing on palliative care in LMIC humanitarian emergency/crisis settings [3, 7]; death, dying, and end-of-life care for refugees, now residing in both high-income countries (HIC) and LMIC settings [8]; end-of-life care in natural disasters, including pandemics in both LMIC and HIC settings [9]; culturally sensitive palliative care in humanitarian contexts, in both HIC and LMIC settings [10]; exploring palliative care for forced migrant families and children now in HIC and LMIC settings [11]; and underrepresentation of palliative care guidelines in infectious disease outbreaks in both HIC and LMIC contexts [12]. None of the systematic or scoping reviews located has specifically explored the palliative care needs of the patient and/or their family in multiple LMIC humanitarian settings as the sole topic, thereby highlighting an apparent gap. Upon reviewing the PROSPERO — International Prospective Register of Systematic Reviews, and the Joanna Briggs Institute Systematic Review Register, there did not appear to be any systematic or scoping review protocols directly related to this specific topic.

This systematic scoping review will provide a current depiction of the palliative care needs of patients and/or their families in LMIC humanitarian settings. This type of review has been chosen as it ensures a broader scope, enabling less restrictive inclusion criteria and the bringing together of evidence from disparate sources [13].

### Clarifying terms/definitions for the purpose of the review

The WHO in 2002 ([14] p. 84) defines palliative care as “an approach that improves the quality of life of patients and their families facing the problem associated with life-threatening illness, through the prevention and relief of suffering by means of early identification and impeccable assessment and treatment of pain and other problems, physical, psychosocial and spiritual.” In 2018, the WHO publication “Integrating palliative care and symptom relief into the response to humanitarian emergencies and crises” outlined how the “prevention and relief of suffering should be made accessible for anyone suffering physically, psychologically, socially or spiritually and not only for those with life- threatening conditions” ([2] p. 17). De Boer et al. [12] outlined how palliative care is for people with chronic or life-limiting illness, and the *Field Manual for Palliative Care in Humanitarian Crises* describes how palliative care has become a distinct subspecialty with the emphasis on care provision for people and their families with severe and life-limiting illness, regardless of the imminence of death [4].

For the purpose of this review, we will define palliative care as the care (outlined in the WHO 2002 definition [14]) provided for the needs of people with serious and/or chronic conditions and/or their families. The use of the term “serious and/or chronic conditions” is reflective of the discussions in the literature outlined above regarding defining palliative care in humanitarian contexts.

Palliative care needs will be defined as “multidimensional problems, symptoms, distress and concerns which can benefit from palliative care” [15, 16]. LMIC humanitarian settings will include public health emergency, acute refugee context, protracted refugee context, and natural disaster, taken from the four sub-studies outlined by the Humanitarian Health Ethics Research Group [17]. We are adding two additional categories — conflict [2, 18] and internally displaced people. As this is a rapidly growing area, a category of “other” will also be included.

## Methods

### Study design

Arksey and O’Malley’s [19] scoping review framework, with additions from Levac et al. [20], will be used to guide the broad outline of the methodology. The five steps from their frameworks will be broadly followed: (1) Identify the research question, (2) identify relevant studies, (3) study selection, (4) charting the data, and (5) collating, summarizing, and reporting the results. Additional methodology from the Joanna Briggs Institute (JBI) [13] and the Preferred Reporting Items for Systematic reviews and Meta-Analyses extension for Scoping Reviews (PRISMA-ScR) from Tricco et al. [21] will also be incorporated to enhance rigor.

#### Stage 1: Identify the research question

The JBI [13] recommended the use of the PCC pneumonic — “participants, concept and context” to guide both the development of the research question and the inclusion criteria. The PCC listed are as follows:

##### Participants

People who have serious and/or chronic health conditions which have the capacity to benefit from palliative care as outlined by the WHO [14], as reported by themselves, their relatives or a health professional, and/or their family members.

##### Concept

The palliative care needs of people with serious and/or chronic conditions and/or their family members.

##### Context

LMIC (including low-, lower-middle-, and upper-middle-income country settings as identified by the World Bank [16]) humanitarian settings including, but not limited to, public health emergency, acute refugee context (including refugee and forced migration, acute (ongoing) conflict), protracted refugee context and natural disaster (various disasters, including earthquake, hurricane, tsunami, famine) [17], conflict [2, 18], and internally displaced people.

##### Research questions

- What are the palliative care needs of people with serious and/or chronic conditions in LMIC humanitarian contexts?
- What are the palliative care needs of the families of people with serious and/or chronic conditions in LMIC humanitarian contexts?

##### Inclusion criteria

Published research/peer-reviewed studies (including, but not limited to, qualitative, quantitative, mixed-methods, case studies,), gray literature (including, but not limited to, unpublished reports, studies, and data obtained following either requests to key informants/experts in the field and/or from pertinent websites) guidelines and textbook chapters, conference abstracts (for both oral presentations and posters), and posters that meet the below criteria will be considered for inclusion:

- Literature reporting individual palliative care needs for patient and/or family (including, but not limited to, symptoms, problems, concerns, and distress in the psychological, physical, social, and spiritual domains of life) which have the capacity to benefit from palliative care as outlined by the WHO (2002) [14] as reported by themselves, their relatives, or a health professional
- Literature relating to serious and/or chronic health conditions, diseases, and injuries, which demonstrate palliative care needs. This can include, but is not limited to, cancers, organ failure (heart, lung, renal, and liver) dementia, and other diseases (such as, but not limited to, HIV, stroke, and neurological diseases) and injuries.
- Date: From January 2012 to October 2022 (due to the dynamic nature of this field, it is envisaged that the majority of the literature will be relatively recent, and a 10-year period would maximize the opportunity to capture relevant data that remains current. Two previous systematic reviews [3, 7] appear to show only a very small amount of data relevant to this study in earlier years).
- Language: English and Arabic (Arabic is included as the second language due to the number of humanitarian crises in Arabic-speaking regions, which may result in Arabic language data — while data will only be extracted from literature in these languages, if literature in other languages is found, this only will be reported/cited but not included in the analysis due to practical issues relating to translation).
- Population: Adults and children with serious and/or chronic health conditions which have the capacity to benefit from palliative care as outlined by the WHO (2002) [14], as reported by themselves, their relatives, or a health professional and/or their families
- Literature from any LMIC (including low-, lower-middle-, and upper-middle-income country settings as identified/listed by the World Bank 2022 [16]) humanitarian setting (including, but not limited to, public health emergency, acute refugee context (including refugee and forced migration), acute (ongoing) conflict, protracted refugee context and natural disaster (various disasters, including, but not limited to, earthquake, hurricane, tsunami, famine), conflict, and internally displaced people).

Development of the inclusion criteria has been guided by Sepúlveda et al. (2002) [22] as cited by Afolabi et al. (2021) [15], Doherty et al. (2020) [23], Nouvet et al. (2018) [3], Amir et al. (2020) [17], WHO (2018) [2], and Krakauer et al. (2020) [18] and a small number of adapted inclusion criteria used by Afolabi et al. (2021) [15].

##### Exclusion criteria

- Studies/literature relating solely to palliative care in high-income country (HIC) humanitarian contexts (i.e., palliative care in Covid in HIC settings)
- Literature relating to palliative care in LMIC settings which are not classified as humanitarian settings (as identified in inclusion criteria)
- Literature in languages other than English and Arabic will be reported/cited but not included in data analysis due to translation issues.
- Papers that only include opinion — editorials and letters

Due to the limited work undertaken in this very new area, plus several key studies reporting on mixed populations of adults and children, literature relating to both adults and children will be included. This will ensure pertinent data is not excluded.

Conference abstracts will also be included to increase the opportunity to capture pertinent data in this novel field. If additional data or clarification is required from included conference abstracts/posters or full-text studies/literature, attempts will be made to contact the authors.

#### Stage 2: Identify relevant studies

The comprehensive strategy will comprise electronic database searches, examination of included text reference lists to identify further resources [13, 19], and searching the gray literature [13].

Databases to be searched are likely to include CINAHL, MEDLINE, Embase, Global Health, Scopus, Applied Social Science Index and Abstracts (ASSIA), Web of Science, Policy Commons, JSTOR, Library Network International Monetary Fund and World Bank (https://library.worldbankimflib.org/), Google Advanced Search, and Google Scholar. Pre-print databases to be searched are as follows: Preprints.org, medRxiv (https://www.medrxiv.org/), and F1000Research (https://f1000research.com/). Existing networks will be utilized [19], including communication to key experts to identify further unpublished reports/studies/data. Relevant websites will also be examined (these can be found listed in Additional file 1).

The search strategy for the majority of databases will combine three sets of terms/key words — palliative care descriptors, humanitarian setting descriptors, and LMIC descriptors, including all LMIC countries as defined by the World Bank [16]. This approach was adapted from the search strategy used by Nouvet et al. [3] for their systematic review of palliative care in humanitarian crises, where palliative care and humanitarian descriptor sets were used. However, following testing, some of the key words/terms were reviewed, aiming to ensure as far as possible that key indicator papers were located, in addition to attempting to reduce the volume of potentially irrelevant literature and to obtain a more manageable number of citations. For example, Ebola was included as a humanitarian descriptor due to the data written on this humanitarian crisis in LMIC; Covid-19 was also included, although combined with palliative care as a search term in the humanitarian descriptors due to the vast amount of literature on this subject alone. Individual words/terms within each set will be separated by the Boolean descriptor “or” with the Boolean operator “and” used to combine the sets. However, for a number of database, pre-print database, and website searches, it is likely that only two descriptor sets will be used (palliative care and humanitarian) with reduced key words, as this approach appeared more effective during preliminary testing. An example of key words/terms can be found in Additional file 2. It is likely that the search strategy will be adapted for each database/website, to enhance specificity, with further filters applied to improve sensitivity of citations retrieved.

The results of a draft search history, undertaken using Ovid MEDLINE database on 30th July 2022, can be found in Additional file 3. There were no language filters used, and the dates in the search were from 2012 to 2022. This search strategy will be adapted for other databases.

#### Stage 3: Study selection

This is an iterative team process involving searching, refinement of the search strategy (if necessary) and reviewing literature for inclusion [20]. Initial searches of databases will be undertaken, with results exported into the EndNote bibliographic system and duplicates removed. Two reviewers will independently review titles and abstracts against the inclusion and exclusion criteria.

If abstracts are not available, full-text data will be reviewed at this stage; it is predicted this will be for a minority of data, probably websites and some gray literature. The number of papers screened and rejected based on title and abstract screening, in addition to the number of papers retrieved for full text screening, will be recorded. Reviewers will meet at the beginning, middle, and end of the title and abstract review stage, to discuss any issues related to study selection and, if needed, to refine the search strategy [20]. Unresolved disagreements will be resolved by a third reviewer [13].

**Fig. 1:**
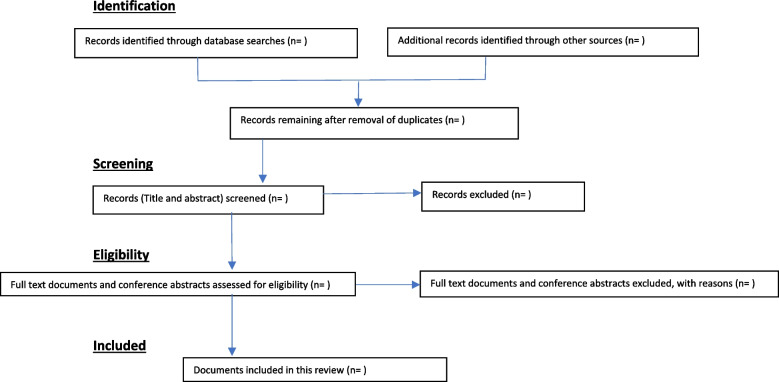
Scoping review flow diagram, showing phases for data extraction and selection (adapted PRISMA-ScR reporting flow diagram, taken from Moher et al. (2009) [28])

Two reviewers will then independently review full-text literature, gray literature, guidelines, textbook chapters, conference abstracts, and posters for inclusion or exclusion, with input from a third reviewer in the event of any disagreements [13, 20] using a table developed by the research team from the eligibility criteria. A summary of this process can be found in Fig. 1.

Reference lists of included documents will be searched, and any relevant abstracts identified will be reviewed following the process outlined above. The websites will also be searched, and any relevant abstracts/data will be reviewed using the process outlined above.

#### Stage 4: Charting the data

**Fig. 2:**
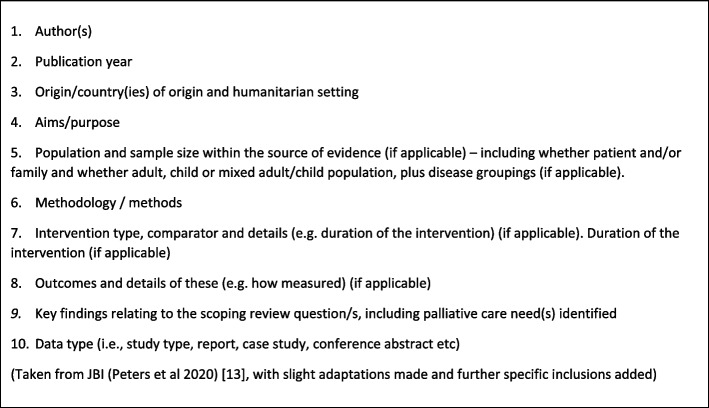
Data extraction chart variables

Data will then be extracted and charted in a table, which aims to provide a summary of the results which is both descriptive and logical [13]. This will be created by the review team collaboratively, ensuring the inclusion of variables to answer the research question(s) [20]. It is currently proposed that this will be developed from a combination of the JBI list [13] with specific pertinent areas added (as outlined in Fig. 2).

Following a pilot of the table [13], data entry will commence, with the chart being updated as agreed by the reviewers, as charting is an iterative process [20]. It is aimed to incorporate both academic and gray literature in the same table, as limited data in this area is predicted.

#### Stage 5: Collating, summarizing, and reporting the results

Extracted data will be initially presented in an overall summary table focusing on the main characteristics of each piece of literature; it is currently planned that this will include origin/humanitarian setting, adult, child or adult/child mixed population, aim, methods, and key findings. Further numerical data, probably relating to literature category, palliative care domain, and humanitarian setting, will be presented visually using tables, diagrams, and/or charts.

Using the principles of framework synthesis, where data in a scoping review is sorted/charted against an a priori framework ([24–26] as cited by 3), it is planned that literature on palliative care needs will then be extracted and categorized into palliative care domain/sub-domain and mapped to a specific humanitarian setting in a table (see table in Additional file 4).

Following the tables and charts, a narrative description will outline how the results relate to the research question(s) [13]. This plan for presenting the results may undergo further revisions during the review process when there is greater awareness of the data available [27].

## Discussion

Following the full search and review, the results will be reported as outlined in stage 5, subject to review depending on the data extracted. It is aimed to publish the review in a peer-reviewed journal, thereby adding to the growing body of evidence on palliative care in humanitarian contexts.

Research ethics approval is not required for this scoping review.

The inclusion of conference abstracts and posters may be a potential limitation, as there is a risk that data may be missing, due to the brevity of the format; authors will be contacted to request further data if possible. However, their inclusion can be justified due to the limited full-text peer-reviewed research in this area.

As patient and family palliative care needs in humanitarian contexts may be a challenging area to identify from article and data titles, literature may be unintentionally omitted; although with the broad search strategy employed in this review, every effort will be made to mitigate this risk.

The inclusion of data relating to both adult and child participants is a strength of this study. The reporting will clearly identify adult, child, or mixed adult/child data (including proportion of adult and child participants where identification is possible).

## Conclusion

This is the first systematic scoping review to specifically explore the palliative care needs of the patient and/or their family, in LMIC humanitarian settings. This format is being utilized to ensure a broad scope is undertaken, enabling the inclusion of literature from disparate sources. This review will form part of a wider research project, exploring palliative care learning needs and evidence-based palliative care curricula for humanitarian health workers, and will contribute to the rapidly growing body of knowledge in this area. It is further anticipated that the findings will highlight research gaps.

### Supplementary Information

**Additional file 1.** Relevant websites to be explored

**Additional file 2.** Examples of key words/terms

**Additional file 3.** Draft search history

**Additional file 4.** Proposed table mapping palliative care needs in relation to domains/sub-domains and humanitarian settings

## Data Availability

Not applicable

## Abbreviations

LMIC: Low- or middle-income country
JBI: Joanna Briggs Institute
PRISMA-ScR: Preferred Reporting Items for Systematic reviews and Meta-Analyses extension for Scoping Reviews
CINAHL: Cumulative Index of Nursing and Allied Health
ASSIA: Applied Social Science Index and Abstracts
HIC: High-income countries
WHO: World Health Organization
PCC: Participants, concept, and context
