# Supplementary material for: Palliative care needs of people and/or their families with serious and/or chronic health conditions in low- or middle-income country (LMIC) humanitarian settings—a systematic scoping review protocol
Source: Syst Rev. 2024 Apr 11;13:105. doi: 10.1186/s13643-024-02521-4 (PMC11007922; doi:10.1186/s13643-024-02521-4)
Supplement: Supplementary file 1 — Additional file 1. Relevant websites to be explored [file 13643_2024_2521_MOESM1_ESM.docx]

**Additional file One:**

**Table and list of relevant websites to be explored**

| **Organisation** | **Website** |
| --- | --- |
| Active Learning Network for Accountability and Performance (ALNAP) | https://www.alnap.org |
| Catholic Relief Services | https://www.crs.org |
| Christian Aid | http://www.christianaid.org/home/1/home.aspx |
| Inter-agency standing committee (IASC) | https://interagencystandingcommittee.org/ |
| International Committee of the Red Cross (ICRC) | https://www.icrc.org/en |
| International Federation of Red Cross and Red Crescent Societies (IFRC) | https://www.ifrc.org/about-ifrc |
| International Rescue Committee | https://www.rescue.org |
| Médecins Sans Frontières (MSF) International | http://www.msf.org |
| United National High Commissioner for Refugees (UNHCR) | http://www.unhcr.org |
| United Nations Office for the Coordination of Humanitarian Affairs (UNOCHA) | https://www.unocha.org/ |
| World Health Organisation (WHO) | http://www.who.int/en/ |

Taken from Nouvet et al (2018 [1]) and DeBoer et al (2021) [2]

In addition to the websites in the above table, further current pertinent websites will be explored, including, but not limited to, Cairdeas International Palliative Care Trust (<https://cairdeas.org.uk/>), Palliative Care in Humanitarian Aid Settings and Emergencies (PalCHASE) <https://www.pallchase.org/>, Humanitarian Health Ethics (<https://humanitarianhealthethics.net/>), ELRHA (<https://www.elrha.org/>), Global Health Academy, University of Edinburgh (<https://www.ed.ac.uk/global-health>), the European Association of Palliative Care (EAPC) <https://www.eapcnet.eu/> and ehospice international edition (<https://ehospice.com/international/>). Furthermore, key journal websites and palliative care association websites will also be searched, to ensure that all opportunity is made to locate pertinent literature.

References:

1. Nouvet E, Sivaram M, Bezanson K, Krishnaraj G, Hunt M, De Laat S, Sanger S, Banfield L, Rodriguez PFE, Schwartz L. Palliative care in humanitarian crises: a review of the literature. International Journal of Humanitarian Action. 2018 ; 3:5

2. De Boer M, Coghlan RJ, Russell B, Philip JAM. The underrepresentation of palliative care in global guidelines for responding to infectious disease outbreaks: a systematic narrative review. International Health. 2021; [doi.org/10.1093/inthealth/ihab075](https://doi.org/10.1093/inthealth/ihab075)
