# Supplementary material for: Palliative care needs of people and/or their families with serious and/or chronic health conditions in low- or middle-income country (LMIC) humanitarian settings—a systematic scoping review protocol
Source: Syst Rev. 2024 Apr 11;13:105. doi: 10.1186/s13643-024-02521-4 (PMC11007922; doi:10.1186/s13643-024-02521-4)
Supplement: Supplementary file 2 — Additional file 2. Examples of key words/terms [file 13643_2024_2521_MOESM2_ESM.docx]

**Additional File Two:**

Example of terms/ key words that may be included:

Palliative OR end-of-life OR terminal care OR palliative care needs OR dying OR bereaved OR bereavement OR grief OR hospice

AND

Humanitarian OR refugee OR armed conflict OR natural disaster OR epidemic OR pandemic OR migrant OR internally displaced OR Ebola OR (covid-19 AND palliative)

AND

Afghanistan OR Albania OR Algeria OR American Samoa OR Angola OR Argentina OR Armenia OR Azerbaijan OR Bangladesh OR Belarus OR Belize OR Benin OR Bhutan OR Bolivia OR Bosnia OR Botswana OR Brazil OR Bulgaria OR Burkina Faso OR Burundi OR Cabo Verde OR Cambodia OR Cameroon OR Central African Republic OR Chad OR China OR Colombia OR Comoros OR Congo OR Costa Rica OR Ivory Coast OR Cuba OR Djibouti OR Dominica OR Dominican Republic OR Ecuador OR Egypt OR El Salvador OR Equatorial Guinea OR Eritrea OR Eswatini OR Ethiopia OR Fiji OR Gabon OR Gambia OR Georgia OR Ghana OR Grenada OR Guatemala OR Guinea OR Guinea-Bissau OR Guyana OR Haiti OR Honduras OR India OR Indonesia OR Iran OR Iraq OR Jamaica OR Jordon OR Kazakhstan OR Kenya OR Kiribati OR North Korea OR Kosovo OR Kyrgyz Republic OR Lao PDR OR Lebanon OR Lesotho OR Liberia OR Libya OR Madagascar OR Malawi OR Malaysia OR Maldives OR Mali OR Marshall Islands OR Mauritania OR Mauritius OR Mexico OR Micronesia OR Moldova OR Mongolia OR Montenegro OR Morocco OR Mozambique OR Myanmar OR Namibia OR Nepal OR Nicaragua OR Niger OR Nigeria OR North Macedonia OR Pakistan OR Palau OR Papua New Guinea OR Paraguay OR Peru OR Philippines OR Russia OR Rwanda OR Samoa OR Sao Tome and Principe OR Senegal OR Serbia OR Sierra Leone OR Solomon Islands OR Somalia OR South Africa OR South Sudan OR Sri Lanka OR Saint. Lucia OR Saint Vincent and the Grenadines OR Sudan OR Suriname OR Syria OR Tajikistan OR Tanzania OR Thailand OR Timor-Leste OR Togo OR Tunisia OR Turkey OR Turkmenistan OR Tuvalu OR Uganda OR Ukraine OR Uzbekistan OR Vanuatu OR Vietnam OR West Bank OR Gaza OR Yemen OR Zambia OR Zimbabwe OR low-income country OR middle-income country
