# Supplementary material for: Palliative care needs of people and/or their families with serious and/or chronic health conditions in low- or middle-income country (LMIC) humanitarian settings—a systematic scoping review protocol
Source: Syst Rev. 2024 Apr 11;13:105. doi: 10.1186/s13643-024-02521-4 (PMC11007922; doi:10.1186/s13643-024-02521-4)
Supplement: Supplementary file 3 — Additional file 3. Draft search history [file 13643_2024_2521_MOESM3_ESM.docx]

**Additional File Three - Draft search history from Ovid Medline (30^th^ July 2022)**

| **Search number** | **Terms and Boolean operators used** | **Hits** |
| --- | --- | --- |
| 1 | Palliative Care/ or palliative.mp. OR terminal care.mp. OR end-of-life.mp. OR "palliative care needs".mp. OR dying.mp. OR bereavement.mp. OR grief.mp. OR hospice.mp. | 65,953 |
| 2 | humanitarian.mp. OR refugee*.mp. OR "natural disaster*".mp. OR migrant*.mp. OR "internally displaced".mp. OR ebola.mp. OR (Palliative Care/ or palliative.mp. AND covid-19.mp.) OR "armed conflict".mp. | 32,080 |
| 3 | Afghanistan.mp OR Albania.mp OR Algeria.mp OR American Samoa.mp OR Angola.mp OR Argentina.mp OR Armenia.mp OR Azerbaijan.mp OR Bangladesh.mp OR Belarus.mp OR Belize.mp OR Benin.mp OR Bhutan.mp OR Bolivia.mp OR Bosnia.mp OR Botswana.mp OR Brazil.mp OR Bulgaria.mp OR Burkina Faso.mp OR Burundi.mp OR Cabo Verde.mp OR Cambodia.mp OR Cameroon.mp OR Central African Republic.mp OR Chad.mp OR China.mp OR Colombia.mp OR Comoros.mp OR Congo.mp OR Costa Rica.mp OR Ivory Coast.mp OR Cuba.mp OR Djibouti.mp OR Dominica.mp OR Dominican Republic.mp OR Ecuador.mp OR Egypt.mp OR El Salvador.mp OR Equatorial Guinea.mp OR Eritrea.mp OR Eswatini.mp OR Ethiopia.mp OR Fiji.mp OR Gabon.mp OR Gambia.mp OR Georgia.mp OR Ghana.mp OR Grenada.mp OR Guatemala.mp OR Guinea.mp OR Guinea-Bissau.mp OR Guyana.mp OR Haiti.mp OR Honduras.mp OR India.mp OR Indonesia.mp OR Iran.mp OR Iraq.mp OR Jamaica.mp OR Jordon.mp OR Kazakhstan.mp OR Kenya.mp OR Kiribati.mp OR North Korea.mp OR Kosovo.mp OR Kyrgyz Republic.mp OR Lao PDR.mp OR Lebanon.mp OR Lesotho.mp OR Liberia.mp OR Libya.mp OR Madagascar.mp OR Malawi.mp OR Malaysia.mp OR Maldives.mp OR Mali.mp OR Marshall Islands.mp OR Mauritania.mp OR Mauritius.mp OR Mexico.mp OR Micronesia.mp OR Moldova.mp OR Mongolia.mp OR Montenegro.mp OR Morocco.mp OR Mozambique.mp OR Myanmar.mp OR Namibia.mp OR Nepal.mp OR Nicaragua.mp OR Niger.mp OR Nigeria.mp OR North Macedonia.mp OR Pakistan.mp OR Palau.mp OR Papua New Guinea.mp OR Paraguay.mp OR Peru.mp OR Philippines.mp OR Russia.mp OR Rwanda.mp OR Samoa.mp OR Sao Tome and Principe.mp OR Senegal.mp OR Serbia.mp OR Sierra Leone.mp OR Solomon Islands.mp OR Somalia.mp OR South Africa.mp OR South Sudan.mp OR Sri Lanka.mp OR Saint. Lucia.mp OR Saint Vincent and the Grenadines.mp OR Sudan.mp OR Suriname.mp OR Syria.mp OR Tajikistan.mp OR Tanzania.mp OR Thailand.mp OR Timor-Leste.mp OR Togo.mp OR Tunisia.mp OR Turkey.mp OR Turkmenistan.mp OR Tuvalu.mp OR Uganda.mp OR Ukraine.mp OR Uzbekistan.mp OR Vanuatu.mp OR Vietnam.mp OR West Bank.mp OR Gaza.mp OR Yemen.mp OR Zambia.mp OR Zimbabwe.mp OR “low-income country”.mp OR “middle-income country”.mp | 725,272 |
| 4 | 1 AND 2 AND 3 | 183 |
