# Supplementary material for: Palliative care needs of people and/or their families with serious and/or chronic health conditions in low- or middle-income country (LMIC) humanitarian settings—a systematic scoping review protocol
Source: Syst Rev. 2024 Apr 11;13:105. doi: 10.1186/s13643-024-02521-4 (PMC11007922; doi:10.1186/s13643-024-02521-4)
Supplement: Supplementary file 4 — Additional file 4. Proposed table mapping palliative care needs in relation to domains/sub-domains and humanitarian settings [file 13643_2024_2521_MOESM4_ESM.docx]

**Additional File Four:**

**Table - Map of palliative care needs in relation to domains/sub-domains (taken from Afolabi et al 2021 [1], WHO 2002 [2] and Dehghan et al 2012 [3]) and humanitarian settings (taken from Amir et al 2020 [4], WHO 2018 [5] with addition of internally displaced category)**

| Palliative  Care needs    Humanitarian  Setting | Physical domain | Psychological domain | | Social domain | | | Spiritual domain |
| --- | --- | --- | --- | --- | --- | --- | --- |
|  | Physical needs | Emotional needs | Information needs | Relationship needs | Financial needs | Practical and support needs | Spiritual needs |
| Refugee acute context |  |  |  |  |  |  |  |
| Protracted refugee context |  |  |  |  |  |  |  |
| Conflict |  |  |  |  |  |  |  |
| Public health emergency |  |  |  |  |  |  |  |
| Natural disaster |  |  |  |  |  |  |  |
| Internally displaced |  |  |  |  |  |  |  |
| Other |  |  |  |  |  |  |  |

References

1. Afolabi OA, Nkhoma K, Maddocks M, Harding R. What constitutes a palliative care need in people with serious illnesses across Africa? A mixed-methods systematic review of the concept and evidence. Palliative Medicine. 2021; 35 (6): 1052-1070

2. World Health Organization. National cancer control programmes: policies and managerial guidelines. 2nd edition. World Health Organization. 2002. [https://apps.who.int/iris/handle/10665/42494 Accessed 30/07/22](https://apps.who.int/iris/handle/10665/42494%20Accessed%2030/07/22)

3. Dehghan R, Ramakrishnan J, Uddin-Ahmed N, Harding R. They patiently heard what we had to say…this felt different to me’: the palliative care needs and care experiences of advanced cancer patients and their families in Bangladesh. BMJ Supportive and Palliative Care. 2012; 2: 145-149

4. Amir T, Yantzi R, de Laat S, Bernard C, Elit L, Schuster-Wallace C, Redwood Campbell L, Hunt M, Schwartz L. ‘Dying alone is hard anywhere in the world’: Palliative care in natural disaster response. 2020 Available at: <https://humethnet.files.wordpress.com/2020/06/natural-disasters-report-and-recommendations.pdf> Accessed on 16/12/21

5. World Health Organization. Integrating palliative care and symptom relief into the response to humanitarian emergencies and crises. 2018. <https://www.who.int/publications/i/item/9789241514460> Accessed 30 July 2021
